# Current Research Status of Respiratory Motion for Thorax and Abdominal Treatment: A Systematic Review

**DOI:** 10.3390/biomimetics9030170

**Published:** 2024-03-12

**Authors:** Yuwen Wu, Zhisen Wang, Yuyi Chu, Renyuan Peng, Haoran Peng, Hongbo Yang, Kai Guo, Juzhong Zhang

**Affiliations:** 1 Suzhou Institute of Biomedical Engineering and Technology, Chinese Academy of Sciences, Suzhou 215163, China; 2 School of Biomedical Engineering (Suzhou), Division of Life Sciences and Medicine, University of Science and Technology of China, Hefei 230026, China

**Keywords:** respiratory motion, image guide, artificial intelligence, machine learning, tumor treatment

## Abstract

Malignant tumors have become one of the serious public health problems in human safety and health, among which the chest and abdomen diseases account for the largest proportion. Early diagnosis and treatment can effectively improve the survival rate of patients. However, respiratory motion in the chest and abdomen can lead to uncertainty in the shape, volume, and location of the tumor, making treatment of the chest and abdomen difficult. Therefore, compensation for respiratory motion is very important in clinical treatment. The purpose of this review was to discuss the research and development of respiratory movement monitoring and prediction in thoracic and abdominal surgery, as well as introduce the current research status. The integration of modern respiratory motion compensation technology with advanced sensor detection technology, medical-image-guided therapy, and artificial intelligence technology is discussed and analyzed. The future research direction of intraoperative thoracic and abdominal respiratory motion compensation should be non-invasive, non-contact, use a low dose, and involve intelligent development. The complexity of the surgical environment, the constraints on the accuracy of existing image guidance devices, and the latency of data transmission are all present technical challenges.

## 1. Introduction

Cancer is a major public health problem worldwide. In 2020, cancer accounted for 18% of all deaths and remained the second-leading cause of death after heart diseases in the United States [1].

GLOBOCAN 2020 reports an estimated 19.3 million cancer cases and 10 million cancer deaths worldwide. Among these total cases, the most common cancers were female breast cancer (11.7%), lung cancer (11.4%), and prostate cancer (7.3%). The main causes of cancer deaths were lung cancer (18%), liver cancer (8.3%), stomach cancer (7.7%), and breast cancer (6.9%) [2]. The top four cancers are located in the chest and abdomen. GLOBOCAN data indicate that East Asia reported the highest number of cases, 6 million (31.1% of the total), with 3.6 million deaths (36.3%). North America reported 2.6 million cases (13.3%), accounting for 7% of cancer deaths, while Central and South Asia reported 1.95 million (10%) and 1.3 million (12.6%) deaths. In Europe, the reported incidence was 4.4 million, of which 1.9 million (20%) died [2]. Early diagnosis and treatment can significantly improve the survival rate of cancer patients. The treatment of cancer includes surgery, radiotherapy, and chemotherapy.

However, during the treatment of thoracic and abdominal diseases, respiratory movement leads to uncertainty in the shape, volume, and location of the tumor, making it difficult to treat in the thoracic and upper abdominal regions. Breathing is the interaction of muscle contraction and relaxation, which increases the chest volume, reduces the pressure, and brings air into the lungs, leading to chest, abdomen, or pelvic periodic motion (in cm) [3]. Virtually all organs in the chest and abdomen are affected by breathing movements, with the lungs being the most affected [4].

During external radiotherapy to targets located in the chest and abdomen, organ deformation changes cause significant geometric treatment errors, which affect tumor control and increase the probability of normal tissue complications [3].

In radiotherapy, to reduce damage to healthy tissues, the radiation beam needs to be delivered accurately to the target area. For tumor movement caused by breathing in the human body, precise radiation therapy is usually achieved by estimating the location of the tumor to ensure that the radiation beam synchronizes with the respiratory movement of the tumor during treatment. Breathing also has a great influence on the timing, accuracy, and injury of a chest puncture. Therefore, the estimation and prediction of respiratory movement plays an important role in ensuring the safety of clinical application, especially in motion compensation.

Radiofrequency ablation (RFA) is an effective minimally invasive treatment for a variety of solid tumor cancers, including those of the lung, breast, kidney, pancreas, and liver. However, effective RFA for abdominal tumors relies on precise ablation needle targeting, which can be a challenging task due to respiratory movement [5].

In thoracic and abdominal puncture surgery, a robotic puncture system (RPS) has the advantages of accurate positioning, flexible movement, and stable operation, and various tissue biopsy diagnosis and surgical scenarios can produce more significant therapeutic impacts. Due to the characteristics of rapid speed, short cycle, and multi-dimensional displacement, respiratory movement may lead to systematic deviation of the target area of thoracoabdominal puncture and affect the therapeutic effect [6].

In angiography, rotary coronary angiography uses the C-arm angiography system to achieve intraoperative three-dimensional imaging, which is considered to be conducive to diagnostic evaluation and interventional guidance. Due to interference with breathing and heart movement in the scan, rotational angiography has not been successfully established in clinical practice for coronary surgery [7].

Extracorporeal shock wave lithotripsy (ESWL) uses an electromagnetic pulse generator to locate stones by X-ray or ultrasound. Respiratory movement may cause X-ray irradiation to deviate from the target area, and to ensure the accuracy of lithotripsy, intraoperative X-ray irradiation of the patient is required. This adds to the complexity of the operation, and the frequent use of X-rays can damage normal tissue [8].

Therefore, the management of respiratory movement is very important for clinical treatment.

One of the most challenging tasks of the radiotherapy robot is how to accurately illuminate a tumor in the chest or abdomen that moves under the action of breathing. In recent years, many technical methods of active motion compensation have been studied, such as a multi-blade collimator and Cyberknife synchronization system (Accuracy Inc.; Sunnyvale, USA), which can automatically track through stereoscopic imaging by placing a small number of reference markers in or near the tumor. However, tracking these benchmarks in real time requires X-ray imaging and the normal tissue receives potentially damaging additional radiation. To overcome this problem, a correlation model is trained, usually using the connection between the interior and exterior of the chest and abdomen [9].

The trajectory of the tumor is strongly correlated with the respiratory movement on the skin surface of the chest and abdomen. Through external respiratory monitoring, the tumor location can be indirectly estimated during radiotherapy [10].

Modern external beam radiotherapy techniques for cancer treatment include three-dimensional conformal radiotherapy (3D-CRT), intensity-modulated radiotherapy (IMRT), helical intensity-modulated radiotherapy (HT), and hadron therapy (HTH). Changes in organ density due to sex and respiratory movements can affect the radiation range and may result in overdoses to critical organs or underdoses to tumors. Therefore, compensation for respiratory movement is crucial [11]. CyberKnife uses three external markers to capture the respiratory characteristics of patients, establish the correlation function between markers and tumor location, and then estimate the tumor location. The location of the tumor and the movement of the tumor are estimated by the markers, and the relevant models are constructed by segmentation of the markers according to the respiratory phase, such as exhaled and inhaled states [12].

The methods of tumor location estimation can be divided into direct estimation and indirect estimation. In terms of the direct estimation of the tumor location, Keall et al. [13] used gold markers implanted in tumors to estimate the tumor location based on current and past X-ray images. Although the real-time location of tumors can be intuitively obtained, long-term exposure to X-ray and gold marker implantation will cause harm to human health. Because ultrasound has the advantages of being non-invasive and not using radiation in medical applications, Huang et al. [14] proposed the application of respiratory tumor motion tracking based on two-dimensional ultrasound images in radiotherapy. Liu et al. [15] used the cascaded single deformant convolutional neural network (COSD-CNN) to continuously extract and track objects in two-dimensional ultrasonic sequences. Pressing the ultrasound probe during treatment may interfere with the patient’s breathing, and since the method was developed for two-dimensional motion tracking, it is not effective in three-dimensional motion tracking.

The accuracy of medical imaging technology makes image-guided surgery an important means of tumor diagnosis and treatment, that is, ultrasound (US), CT, magnetic resonance imaging (MRI), and other medical images guide treatment operations on the target area. Based on chest and abdominal CT images, Danielle F. Pace et al. proposed a local adaptive regularized deformation image registration method of sliding organs based on anisotropic diffusion [12], and Matthew J. et al. used four-dimensional cone-beam computed tomography (4D-CBCT) data with deformable grouping registration to perform respiratory motion compensation [16]. For MRI images, V. Hamy et al. studied the application of dynamic MRI respiratory motion correction based on robust data decomposition registration [17]. Using DCE-MRI, P. Wan et al. studied transmission-based liver ultrasound compensation for irregular respiratory motion [18].

Researchers at home and abroad have conducted in-depth and extensive research on the technique of intraoperative respiratory motion compensation and achieved fruitful research results. The respiratory motion compensation technique has important clinical value in solving the problem of inaccurate tracking of target area caused by respiratory motion in thoracic and abdominal surgery. At present, some chest and abdominal hand robots equipped with respiratory monitoring and prediction technology have produced commercial products, but there are still problems, such as normal tissue damage and long delays. There are still many safety hazards caused by respiration in radiotherapy, thermal ablation, punctures, and other operations, and there are many key technical and scientific problems that need to be solved. All parts of this paper are organized as follows: direct tracking methods through respiratory monitoring, respiratory simulation and prediction based on indirect models or digital phantoms, and existing research techniques for prediction of respiratory movement through learning are introduced. The discussion part discusses and analyzes the existing research on the above three types of methods, and finally points out the development direction and challenges of chest and abdominal respiratory movement monitoring and tracking.

## 2. Methods

### 2.1. Data Sources and Search Strategy

A chronological review of several reputable literature databases, namely, Science Direct, IEEE, Springer, Nature, and Google Scholar, was conducted to identify trends in the prediction and monitoring of respiratory motion in thoracic and abdominal therapy from 2013 onward. The keywords used to examine more classified papers were “Respiratory compensation” and “Respiratory motion”.

The search results were further filtered using the keywords “thoracico-abdominal”, “tumor tracking”, “radiotherapy”, and “surgery”. A set of inclusion and exclusion criteria was then applied to select the appropriate literature.

### 2.2. Inclusion and Exclusion Criteria

The following inclusion and exclusion criteria were used to screen out discrete knowledge critical to the systematic review and temporal review of respiratory motion for thorax and abdominal treatment.

Inclusion criteria:

- The paper introduced respiratory compensation and prediction in thoracic and abdominal surgery, including image-guided radiotherapy, thoracocentesis, or respiratory monitoring.
- The paper was available to the authors and was a scientific article written in English.
- The device or method considered was used to solve respiratory motion interference during treatment.

Exclusion criteria:

- The device or method was originally intended for use on parts of the body other than the thorax and abdomen.
- The study only evaluated system performance or clinical trials, with a lack of information in terms of design.

A framework was developed to analyze the relatively large number of studies found in the literature search. The aim was to extract the various techniques used in the developed devices and present them in a logical and systematic order so that future designers can gain knowledge of existing methods.

The literature was classified according to respiratory compensation methods, which was divided into direct tracking, indirect model-based respiratory prediction, and indirect learning-based respiratory prediction, and was subdivided according to whether the imaging mechanism directly contacts the human body or guides the image.

Finally, the research progress of the respiratory compensation scheme in thoracic and abdominal surgery in recent years was summarized. While the framework does not address the specific building process of the device, its potential lies in the various building blocks needed to design the device and the methods that have been explored.

## 3. Results

### 3.1. Direct-Tracking Methodology

The methods for directly tracking thorax and abdomen respiratory motion have made great progress in the past 10 years, and the main research institutions include the University of Lübeck, University of Bourgogne Franche-Comté, Friedrich-Alexander-Universität Erlangen-Nürnberg, Soochow University, and Shandong University.

The accurate assessment and quantification of respiration-induced target motion and its integration into the treatment workflow are essential for adaptive treatment techniques. This section is divided according to whether or not the tracking technology is directly in contact with the body [19].

The overview of tracking strategies for respiratory motion is shown in Table 1.

#### 3.1.1. Contact Methods

Respiratory monitoring is required in many surgical settings, often with the help of optical or infrared sensor devices.

In 2016, A. Andreychenko et al. [20] from the University Medical Center Utrecht developed a passive breathing motion sensor based on the noise variance of the receiver coil array. Due to respiratory motion changing the resistance of the body, the RF coil noise variance depends on the resistance of the body, and thus, the respiratory modulation. The depth of noise variance modulation due to breathing varies between the individual channels of the array and depends on the position of the channel relative to the body, with a maximum modulation restriction of 3% for monitoring normal breathing. The noise sensor combined with MR acquisition can detect the breathing movement of each K-space read line. In a clinical MR system, the noise in the receiving array can detect respiratory movement. In contrast with breathing belts, noise sensors do not require careful positioning, any additional hardware, and/or MR acquisition.

Passive monitoring of thermal noise changes in the receiving array channel shows the respiratory movement of the underlying anatomy, i.e.; a so-called “noise navigator”. However, due to the passive nature of noise navigators, there is an inevitable trade-off between accuracy and temporal resolution. A time filter must be added to the noise navigator to accurately display respiration and maintain the time resolution. For real-time applications of noise navigators, such as prospective motion correction or motion tracking, the added filters must be prospective. Thus, in 2018, Navest R. J. M. et al. [21] from University Medical Center Utrecht continued to design a prospective Kalman filter to predict breathing from the noise navigator. The breathing signal can be measured by the noise navigator independently of MR acquisition. A strong linear relationship was found between the anticipatory noise navigator and the quantitative 2D image navigator for measurements, including free breathing and task breathing.

With the rapid development of image-guided surgery, intraoperative guidance may be of great benefit in ensuring radical resection margins in cancer surgery. Kok E. N. D. et al. [24] used the NDI Aurora V2 Electromagnetic (EM) tracking system (Northern Digital Inc.; Waterloo, ON, Canada) to connect pre-operative image data to the intraoperative patient settings. A tracker with an EM sensor was used to determine the patient’s position during surgery, and a tracking sensor was placed on the tumor to adjust for the real-time tumor movement. Through navigation software, the acquired images were registered with preoperative diagnostic protocol CT scans, enabling real-time tumor tracking with a median target registration accuracy of up to 3 mm.

The CyberKnife subsystem Synchrony respiratory tracking system was the first technology that can continuously synchronize beam delivery with tumor motion [37]. External breathing movements are tracked using three optical reference markers attached to a body vest, with small gold markers implanted near the target area to ensure a continuous correspondence between internal and external movements. Varian’s Calypso prostate motion tracking system builds internal–external motion modeling by implanting three tiny sensors and associated wireless tracking [38]. The BrainLAB ExacTrac positioning system uses radioactive opaque markers implanted near a center, such as a target, and is equipped with external infrared (IR)-reflective markers [39]. Internal markers are tracked by an X-ray positioning system, while infrared stereo cameras track external markers. The Xsight lung tracking system is a respiratory movement tracking system for lung lesions without the need to implant reference markers [22,40].

In 2016, Wijenayake Udaya and Park Soon-Yong et al. [22] from Kyungpook National University used an RGB-D camera and principal component analysis (PCA) to track and model the feasibility of external breathing movements for a specific individual. Marker-based depth frame registration technology has also been introduced to limit the measurement area to an anatomically consistent area during treatment. The accuracy of the proposed method was evaluated using a breathalymeter and a laser line scanner. Four white dot markers were used to define the measurement boundaries of the moving chest wall, providing a consistent area for the estimation of respiratory movement. Continuous depth images of breathing movement were captured using an RGB-D camera. Principal component analysis (PCA) was applied to these depth data to break down the breathing motion into a set of motion bases corresponding to the principal component (PCs). Through the analysis of multiple data, they found that only the first principal component could accurately capture the breathing motion, and to evaluate the motion in the metric space, the depth image was reconstructed using a projection coefficient while removing the noisy data represented by a smaller PC. Using the advantages of RGB-D cameras, they proposed a new method for tracking breathing movement that achieved a high correlation (0.99) between the method and the laser line scanner, with an average motion error of 0.76 mm [41].

In 2020, Y. Yu et al. [8] from Shanghai Jiao Tong University proposed a respiratory monitoring system based on Aruco, which is an open source augmented reality library for reference marker tracking. Aruco relies on black and white markers that can be located by image recognition codes to optimize the mark detection using digital image processing algorithms and convolutional neural network (CNN)-based methods.

M. Musa et al. [23] at the University of Arkansas presented the design, fabrication, and bench characteristics of a patient-fixed, respiration-compensated robotic needle insertion platform. The position error and direction error of isolated pig liver were 1.22 ± 0.31 mm and 1.16° ± 0.44°, respectively. The ablation needle was automatically inserted into the liver tumor during the resting phase of the respiratory cycle. The patient’s respiratory cycle was gated using a GE D690 PET/CT scanner with Varian CT’s real-time location management system. The real-time position management system consists of a reflector attached to an external marker on the patient’s abdomen, which is used to reflect the patient’s breathing pattern. An alternative breathing signal can be captured by an external camera at a frequency of 30 Hz [42]. Even with sudden changes in breathing rate, the system is able to track the real-time position data. Static phantom positioning experiments showed that the position error was 1.14 ± 0.30 mm and the direction error was 0.99° ± 0.36°.

Mechanical ventilation is a life-saving treatment for patients in intensive care units. Unfortunately, mechanical ventilation itself can increase patient morbidity and mortality due to ventilator-induced lung damage [43,44]. Therefore, pulmonary function monitoring and internal respiratory system pressure assessment play a key role in reducing this problem [44,45,46]. In 2019, T. Addabbo et al. [25] at the University of Siena proposed a technique for estimating pleural pressure in respiratory monitoring by combining information from the central venous pressure (CVP) and an electrocardiogram (ECG) signal. Since mechanically ventilated patients should have a correct central venous catheter position, CVP filtration technology can be a useful clinical method to estimate pleural pressure, thus avoiding the use of other invasive devices (e.g.; the characteristics of the proposed measurement method were discussed through theoretical modeling, numerical analysis, and experiments based on signal acquisition in intensive care units. The results confirm the validity of the proposal.

In radiotherapy, by measuring respiratory airflow and chest movement to calculate complex internal respiration and tumor movement, H. L. et al. [26] proposed a method for tracking lung tumors based on a patient-specific biomechanical model that takes into account the physiology of respiratory movement to simulate true, non-repeatable movement. The behavior of the lungs is directly driven by the simulated action of the breathing muscles, namely, the diaphragm and intercostal muscles (thorax). The lung model is monitored and controlled through a personalized lung pressure/volume relationship throughout the respiratory cycle. The lung pressure and rib movement are patient-specific and obtained by alternative measurements. The rib displacement corresponds to the transformation from end-expiratory (EE) to end-inspiratory (EI), which is calculated using the finite helical axis method. The lung pressure is calculated using an optimized framework based on inverse finite element analysis by minimizing the lung volume error (the error between the respiratory volume of the respiratory flow exchange and the simulated volume of the biomechanical simulation calculation). At all stages of breathing, the developed model was able to predict lung tumor movement with a mean landmark error of 2.0 ± 1.3 mm.

**Figure 1:**
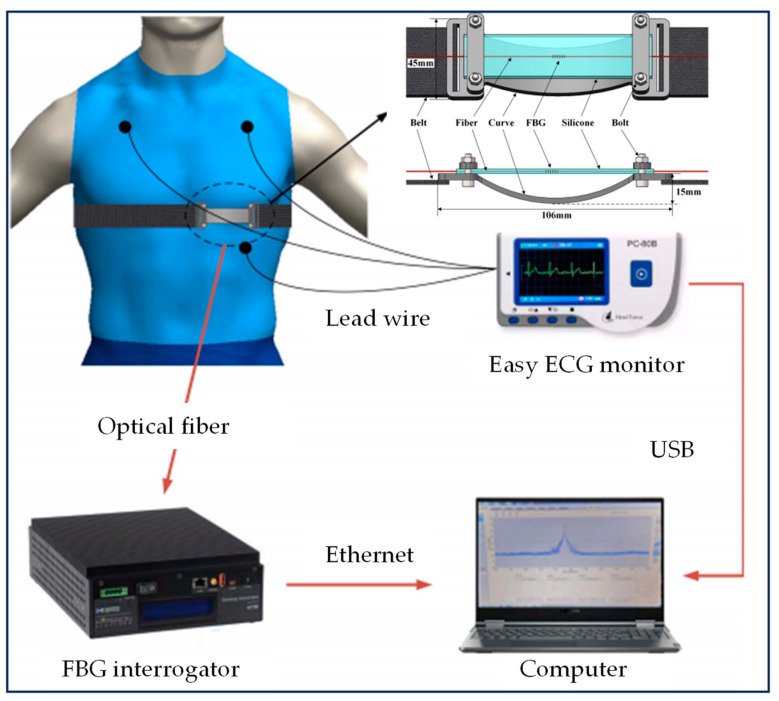
Schematic diagram of a wearable breathing and heartbeat [28].

Respiratory movement has an effect on Fiber Bragg grating (FBG) sensors, and several organs (e.g.; lungs, liver, pancreas) may exert strain on the grating due to breathing-induced movement [47], as shown in Figure 1. Smart textiles based on optical fiber sensors have shown promising results in respiratory monitoring and magnetic resonance (MR) environmental applications. In 2016, C. M. et al. [27] designed and fabricated a six-FBG-based smart textile for monitoring partition volume change, global volume change, and respiratory frequency. Four healthy volunteers were optimized for FBG positioning using a label-based optoelectronic system (OS). The results based on chest wall movement were analyzed by a marker motion capture system. Over time, the proposed textiles showed excellent performance in non-invasive monitoring of zoning and global volume parameters and were compatible with MR. Tested on two volunteers, the system neither produced any image artifacts nor caused discomfort to the volunteers.

In 2023, C. Shi et al. [28] at Tianjin University introduced a new type of wearable sensor based on an FBG, which has high sensitivity and can achieve accurate simultaneous measurement of breathing and heartbeat activity, as shown in Figure 1. The sensor consists of an elastic curved structure, a suspended silicone membrane, an optical fiber engraved with a 3 mm length FBG sensor, and a wearable elastic belt. The sensor structure offers significant advantages in terms of high sensitivity, excellent flexibility, and compactness, making it suitable for wearable device design.

**Figure 2:**
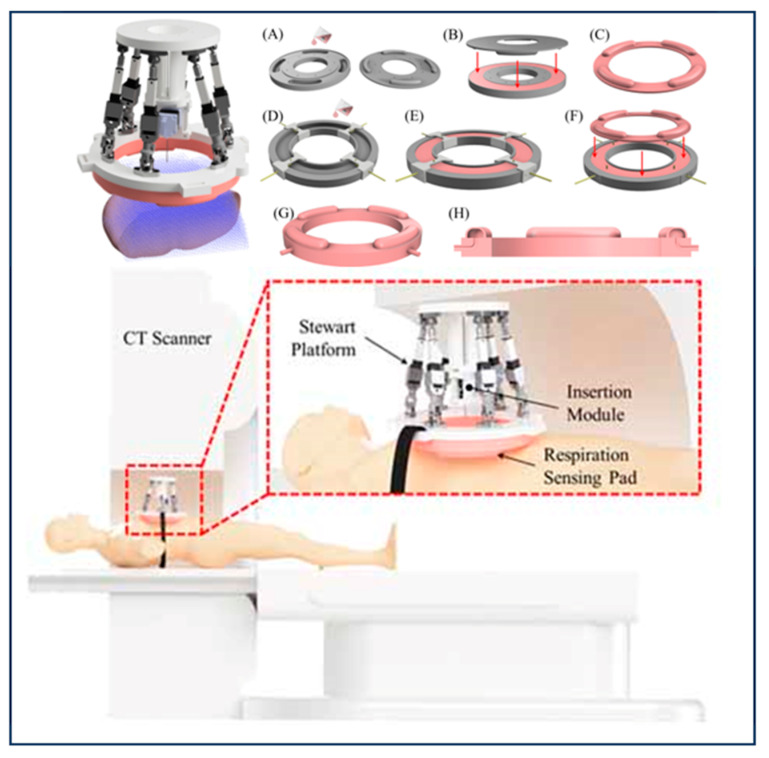
Schematic diagram of breathing sensor pad for abdominal radiofrequency ablation robot: (**A**) pour the silicone into the first mold; (**B**) then place the second mold concentric on the first mold to form a pressure sensing cavity; (**C**) remove the bottom formed part from the mold; (**D**) the third mold is then assembled with the internal pneumatic pipe and the silicone is poured; (**E**) silicone solidifies around the pipe; (**F**) then pour in a thin layer of silicone and place the first molded part over the second molded part; (**G**) the finished product is removed from the mold; (**H**) the inner channels can be seen after the molding process.

In boiling histotripsy treatments, Gilles P. L. et al. [36] from the University of Washington Thomas proposed a practical and economical method for the compensation of unidirectional respiratory motion for the evaluation of BH in isolated tissues. The BH transducer was fixed to a robotic arm and follows the movement of the skin, which is tracked using an inline ultrasound imaging probe. In order to compensate for the system lag and obtain more accurate compensation, an autoregressive motion prediction model was implemented. A BH pulse gating was also implemented to ensure positioning accuracy. The evaluation of in vitro BH therapy was achieved using tissue samples that simulated respiratory movements. The results show that during the course of treatment, the value of the target positioning error was reduced by 89%, while the treatment time increased by no more than 1%. Anthony L. et al. [5] at the Georgia Institute of Technology introduced the design, manufacture, modeling, and bench characteristic testing of a CT-guided parallel robot, and proposed a new breathing motion compensation protocol (RMCP) for the accurate positioning of robot-assisted abdominal RFA needles, as shown in Figure 2. The robot consists of a Stewart platform and a friction-driven drum insertion module that uses a custom-designed breath-sensing pad for motion gating, eliminating the need for continuous CT motion gating. The breathing pad is a molded silicone pressure-sensing device that uses silicone molds to make rubber, ensuring patient comfort when the robot is placed on the patient. The breathing sensing pad unit is connected to the pressure sensor through a pneumatic tube, and when breathing occurs, the abdomen expands and a breathing-related deformation occurs in the pressure unit. This deformation results in an increase in pressure within the unit, resulting in an RMCP. Strain energy models are used to predict the needle insertion force required to effectively penetrate the skin. The mean error of these models was 0.49 ± 0.28 N.

#### 3.1.2. Non-Contact Methods

Tumor tracking allows for continuous radiation dose delivery by dynamically adjusting the radiation beam so that it follows real-time tumor movement. For either technique to be effective, precise measurements of breathing signals are needed. The traditional methods of respiration measurement have problems of invasiveness and low accuracy. Benchmark-based measurements require an invasive implantation procedure with serious risks for patients [48]. External respiratory substitutes can be measured using infrared reflection markers, spirometers, or pressure bands to infer internal tumor locations based on point measurements or numerical indicators that provide respiration [49]. These devices must be in close contact with patients to work, often causing discomfort [29].

C. Gu et al. [29] from Texas Tech University put forward a kind of DC-coupling continuous wave radar sensor, which is used to provide a non-contact measurement that does not hinder breathing. The radar sensor uses a DC-coupled adaptive tuning architecture, including RF coarse tuning and baseband fine tuning, to accurately measure motion and always operate at maximum dynamic range. The accuracy of the respiration measurement of the proposed radar sensor was evaluated experimentally by using physical models, human subjects, and mobile platforms in a radiotherapy environment. The results show that it is feasible to use the radar sensor to measure respiration when the radiation beam is opened, and the measurement accuracy reached the sub-millimeter level.

A structured light system is an optical measurement method with the advantages of being non-contact, allowing full-field measurement, and having a high spatio-temporal resolution. Shi H. Lim et al. [30] at the Universiti Kebangsaan Malaysia used Microsoft’s Xbox Kinect^TM^ to track breathing movements in 2014. The consistency of the respiratory movement was analyzed through the recorded 3D movement data. A graphical user interface (GUI) was developed to display statistical information about respiratory signals and data.

In 2018, P. Hou et al. [50] at Soochow University proposed a method to establish a correlation model between a tumor and a chest and abdomen surface model based on a three-dimensional point cloud, which more fully reflects the correlation between three-dimensional surface information and tumor movement. A preliminary study was conducted on body surface modeling, establishing the correlation model between the tumor and the chest and abdomen surface modeling, and comparing the effects of two modeling methods, namely, point cloud data modeling and external marker modeling, which verified the feasibility of using point cloud data modeling instead of external marker modeling.

In 2020, Andrew L. Fielding et al. [31] investigated the applicability and performance of the Intel RealSense^TM^ D415 depth camera as a tool for measuring respiratory movements on the body’s surface. The accuracy of the camera depth data was characterized by the measurement distance, which ranged from a stationary surface to 1.2 m. The delay of the camera system was also measured. For stationary measurements, the average standard deviation of the depth data was less than 0.2 mm for the shortest distance between the camera and the surface at 400 mm, and 3 mm for distances of 1200 mm. Although the data were noisy, the camera was able to measure deformable breathing motion models with variable surface motion amplitudes between 1.5 and 2.5 mm. The RealSense^TM^ system measured a delay of 68.6 milliseconds ± 9.6 milliseconds. The results show that the D415 RealSense^TM^ depth camera was capable of measuring the external breathing type movement of irregular surfaces. M. Liu et al. [51] evaluated the accuracy of a 3D surface imaging system (Sentinel) in breast cancer patients receiving BCS. The results show that optical surface imaging could be accurately applied in the localization of breast cancer patients without the need for unnecessary imaging doses.

Based on the optical system, L. Zheng et al. [32] at the South China University of Technology proposed a control strategy for a puncture robot to compensate for tissue deformation under clinical breathing and a respiratory tracking and compensation system (RFRS) that autonomously analyzes breathing movement signals, plans an optimized path, and controls the robot to follow the target. Through the real-time analysis of the breathing movement, the movement of the robot arm consists of tracking compensation related to the breathing movement and insertion into the target.

The optical tracking system (OTS) and the robotic arm are responsible for respiratory motion detection and compensation, respectively. The control strategy is divided into two parts: tracking according to the breathing movement and entering the planned target area. During insertion, the robot drives the surgical instrument to the target position. Otherwise, it switches to tracking breathing movements.

The online target positioning model (OTLM) is designed to limit the overlap of surgical tools with planned targets and improve the accuracy, safety, flexibility, and agility of the puncture process [32].

An intraoperative image guide can be used to ensure accurate tracking of the tumor in the operation. Most of these systems use 4D X-ray computed tomography (CT) or magnetic resonance imaging (MRI) techniques to extract respiratory motion.

In 2014, S. Vijayan et al. [33] evaluated the accuracy of motion tracking based on the deformable registration of 4D ultrasound images. A non-rigid registration algorithm designed to estimate motion from dynamic imaging data is used. The method registers the entire 4D image data series in a group-optimized manner, avoiding bias against a specific selected reference point in time. The estimated error of liver motion by this registration method was 1 mm (75% quantile across all datasets), which was 1.4 mm lower than the inter-observer variability. When the time resolution was reduced by a factor of eight, the registration error increased to 2.8 mm.

In 2015, L. R. et al. [34] also proposed a method for tracking deformable anatomical targets in 3D ultrasound imaging, estimating the deformation caused by the physiological motion of the patient. The displacement of the moving structure is estimated by an intensity-based method combined with a physical model, which has the advantage of being insensitive to image noise.

In 2016, J. S. et al. [35] at Stanford University introduced a robotic 4D ultrasound (US) imaging system capable of concurrent radiotherapy beam delivery and estimated the proportion of robotic US image guidance that can be used in stereotactic ablative body radiotherapy (SABR) to the liver without interfering with clinically used VMAT beam configurations. The image-guiding hardware includes a 4D ultrasound machine, an optical tracking system to measure the ultrasound probe attitude, and a custom-designed robot to obtain a hands-free ultrasound volume. By simulating the US propagation using planned CT, the placement of robot US hardware is guided by presenting a target visibility map on the CT surface. The results showed that for PTV targets, the robot US guide could image without mechanical interference 80 percent of the time and was guided without beam interference 60 percent of the time. For smaller GTV targets, these percentages were 95% and 85%, respectively.

Several approaches have emerged to achieve respiratory movement tracking that do not require invasive surgery or patient contact.

### 3.2. Respiratory Prediction Method Based on Indirect Model

Great progress has been made in lung cancer radiotherapy technologies, such as respiratory gating [52,53], breath-holding/control [54,55], and real-time tumor tracking [56]. Although significant advances have been made in the field of radiotherapy in recent years, especially in four-dimensional (4D) imaging, there is an increased risk of treatment complexity and uncertainty in the management of motion. Therefore, it is important to have an appropriate evaluation model. Different forms of motion phantograms have been developed and used to study 4D imaging and 4D radiation dose delivery. These phantoms can be broadly divided into three categories: physical phantoms [57,58], physiological phantoms [59,60], and digital phantoms [61,62,63,64]. Physical phantoms are usually made up of mechanical and electrical components that simulate tumors and body anatomy. Examples include the dynamic Chest Phantom from CIRS (CIRS Inc.; Norfork, VA, USA) and the breath-gated platform from Standard Imaging Inc.; Middleton, WI, USA. A mechanical phantom has a high manufacturing cost, is not realistic enough, and there is a big gap with the real situation. Physiological apparitions are implanted in natural specimens, such as pig lungs, to simulate breathing movements close to the conditions in the body, which can be controlled by a water pump to expand or contract the lungs. Physiological phantoms can measure anatomical parameters, but here the movement-monitoring data of animals is not directly applicable to humans. Physical and physiological phantoms fail to take into account patient anatomy and respiratory biomechanics, and therefore, provide less-than-ideal guidelines for actual treatment.

A digital phantom, which is a computer-based simulation of real human breathing that provides a virtual model of anatomy and physiology, has begun to be used to develop and test imaging and therapeutic technologies. Using accurate computer models of the physical imaging process, imaging data can be generated to accurately simulate a real patient. In treatment planning or image guidance, a digital phantom can compensate for image motion to improve the image quality [65] and evaluate treatment strategies by simulating the therapeutic effects of free breathing [66,67,68].

Respiration prediction can transform the time series prediction problem. The traditional respiratory prediction method is based on historical data to predict the future of sequence data.

Based on image-guided methods, model-based respiration prediction is achieved through the registration of sliding organ deformation images, such as using biomechanics or local adaptive regularization based on anisotropic diffusion [12,69].

Since the respiratory prediction method based on an indirect model is to build a model through the correspondence between historical guidance data and predicted sequence data, this section is divided into X-ray images (including CT, CBCT, and PET), MRI images, ultrasound images, and four other aspects according to the types of guidance data.

#### 3.2.1. X-ray Imaging

**Figure 3:**
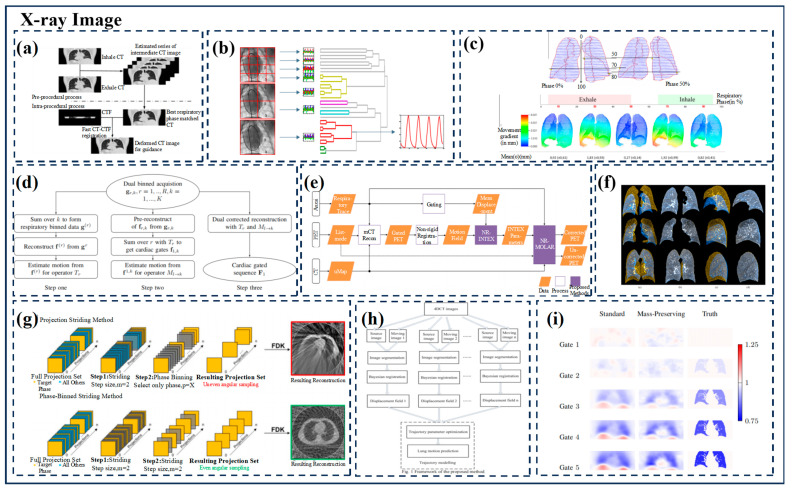
Overview of indirect model respiration prediction based on X-ray images: (**a**) rapid CT and CT fluoroscopy registration for image-guided pulmonary intervention based on respiratory motion compensation; (**b**) unsupervised learning robust respiratory signal estimation from X-ray fluoroscopy; (**c**) respiratory correction 4D reconstruction of gated myocardial perfusion based on SPECT; (**d**) model reconstruction of non-rigid continuous breathing motion compensation list based on PET; (**e**) the regularization key points based on CT correspond to the estimation of lung mass motion with intensive deformation registration; (**f**) based on CT, this can be customized to account for breathing for the development of 4D numerical chest film; (**g**) respiratory motion compensation was driven by 4D-CBCT data with grouped deformable registration; (**h**) prediction of lung motion from 4DCT images using Bayesian registration and trajectory modeling; (**i**) combined with lung density changes to improve PET/CT respiratory motion compensation.

The overview of indirect model respiration prediction based on X-ray images is shown in Figure 3.

CT fluoroscopy (CTF) is a highly efficient imaging technique used to guide percutaneous pulmonary interventional procedures, such as biopsy and ablation, that provides near-real-time feedback on the patient’s anatomy, enabling physicians to make adjustments as they push the needle toward the target lesion. In 2013, P. Su et al. [70] proposed a fast CT-CTF deformable registration algorithm to achieve 3D guidance by deforming the CT image before aspiration into the intraoperative CTF image. In this algorithm, the deformation of the transverse plane is modeled using 2D B-Spline, and the deformation along the z-direction is normalized by smoothness constraints. The respiratory motion compensation (MC) framework is combined to achieve accurate registration. Electromagnetic (EM) tracking provides 3D image guidance during breath-holding.

J. Cai et al. [68] from Duke University developed a computer program to facilitate the characterization and implementation of XCAT phantoms in 4D radiotherapy applications. For an XCAT phantom with a pixel size of 2 mm, the overall mean (±standard deviation) difference in motion amplitude between the input trajectory and the measured trajectory was 1.19 (±0.79) mm. The 4D-CT and 4D-CBCT images based on XCAT Phantom were validated for normal breathing patterns. P. Fischer et al. [71] proposed a new method for extracting respiratory signals from X-ray fluoroscopic images based on unsupervised learning. The perspective images have different and smaller fields of view, C-arm angles, and AECs. This method uses a patch-based approach to extract multiple breathing signals from each image. Respiratory signal extraction uses two unsupervised learning methods, namely, kernel-PCA-based reduction and clustering, where the proposed signals of all patches are clustered to find respiratory information, and the information of all patches is combined to tolerate outliers. The use of respiratory models is beneficial to radiation protection and reduces the dose of radiation. Combined with the movement information required for treatment, the dose delivery is monitored through the patient’s respiratory cycle. P. E. Leni et al. [72] proposed a method based on artificial neural networks (ANNs) to simulate the real breathing movement of the lungs and develop a 4D numerical chest phantom with customizable breathing.

In 2017, W. Q. et al. [73] investigated a 4D reconstruction method used to reduce the effects of respiratory movement in SPECT images of the heart. In this method, heart-gated image sequences are reconstructed according to the reference breathing amplitude box in the breathing cycle. The inherent challenge of high imaging noise is overcome by counting data acquired throughout the breathing cycle. To eliminate intra-cycle and intra-weekly motion during dynamic imaging, C. Chan et al. [74] at Yale University performed a non-rigid breathing motion correction for each event of static and dynamic PET data, and we developed a solution. The continuous deformation field of each voxel is estimated using non-rigid INTEX (NR-INTEX) with a time resolution that matches the external respiration tracking. Non-rigid motion correction was carried out according to the deformation system matrix by non-rigid MOLAR (NR-MOLAR). For a wide range of respiratory movements, J. R. et al. [75] proposed a novel registration algorithm for lung CT scanning by integrating sparse key-point correspondence into a dense continuous optimization framework. This method is mainly divided into two steps: robust sparse corresponding field calculation for a moderate number of key points and continuous optimization deformable registration based on the strength-driven integration of key point correspondence and volume change constraints. The detection of key-point correspondences is robust to large deformations through joint optimization over a large number of potential discrete displacements, while the dense continuous registration achieves subvoxel alignment through smooth transformations. Curvature regularization and volume change control mechanisms are used to prevent the folding of the deformed mesh.

In 2018, M. J. Riblett et al. [16] combined group deformation image registration and motion compensation image reconstruction algorithms to improve the image quality of 4D-CBCT under clinically relevant image acquisition conditions. Group registration is a method of registering all time frames of a 4D image to a common reference frame, which minimizes the impact of any single time point on the global smoothness or accuracy of the deformable model. The 4D cone-beam CT (4D-CBCT)-reconstructed images are registered to iteratively calculated average-frame or fixed-frame reference images to model breathing movements. The resulting 4D transform is used to deform the projection data during Feldkamp–Davis–Kress (FDK) backprojection operations to create motion compensation reconstructions.

M. Li et al. [76] proposed a new method for Bayesian registration and trajectory modeling based on film four-dimensional computed tomography (4DCT) images. Specifically, the method uses CT images captured at the end of inspirations as source images and CT images captured at other stages as moving images. The source image is then aligned with each moving phase image, and the displacement field is generated using a Bayesian registration method. Then, by connecting discrete phase displacement fields, a lung motion trajectory model based on continuous time-dependent displacement fields is established. The results show that the method can accurately predict any point in the lung at any given time.

Then, X. Bao et al. [77] proposed a Bayesian-based PCA statistical model whose estimated accuracy follows the probability distribution associated with the model parameters. Combined with Bayesian probability reasoning, the prior probability was estimated by the preoperative statistical model, and the likelihood ratio was constructed according to the similarity between the intraoperative abdominal surface and the preoperative CT surface. Therefore, the posterior probability of the current internal respiratory motion vector field can be obtained. By maximizing the posterior probability, the best PCA statistical model parameters can be obtained, and then an estimate of the internal respiration motion with the greatest posterior probability can be obtained. The mean error of the model motion estimation was 0.57 ± 0.06 mm when using single-period CT data and 1.52 ± 0.41 mm when using dual-period CT data.

In 2019, to restore the ideal deformation field between chest images containing sliding and smooth motion patterns, L. Gong et al. [78] proposed a regularization term called locally adaptive total P-variation (LaTpV) and embedded it into a parametric registration framework to accurately restore lung motion. LaTpV adaptively balances the smoothness and discontinuity of the displacement field, is suitable for sliding motion correction, and has potential clinical application in the adjustment of radiotherapy schedule.

In 2020, E.C. Emond et al. [79] proposed an experimental framework for combined PET/CT image reconstruction and motion estimation, in which PET images and motion are estimated directly from raw data. The change in volume is estimated by using the “Jacobian determinant” to calculate the deformation field, the problem of density changes during respiration can be taken into account, and the image registration that maintains quality can be directly applied to the joint estimation of PET active images and motion.

#### 3.2.2. MRI

Respiratory motion correction in dynamic contrast-enhanced magnetic resonance imaging (DCE-MRI) is challenging because rapid intensity changes can affect common (intension-based) registration algorithms. In 2014, V. Hamy et al. [17] introduced a novel registration technique based on robust principal component analysis (RPCA) to decompose a given time series into low-rank and sparse components. RPCA combined with residual complexity minimization registration algorithm can accurately register a DCE time series for various organs and different respiratory protocols. Because of the clear isolation of a sparse term, RPCA should be more flexible and robust than conventional principal component analysis and may contribute to DCE-MRI registration. The algorithm reduces the error in organizing time–intensity curves by 15–62%.

In order to improve the accuracy of interventional catheter guidance in cardiac surgery and solve the problem where accuracy is limited by respiratory movement, R. Xu et al. [19] proposed to establish a respiratory movement model that compensates for errors under the guidance of magnetic resonance imaging (MRI) in 2015. The 2D real-time free-breathing images were collected to characterize the breathing movement, and then the previous 3D images were registered with the real-time images in the anatomically relevant frame of reference (FOR) of the spindle to establish a smooth movement model. Combined with real-time imaging data, it provides high temporal resolution and can simulate respiratory motion more accurately. Experimental results show that this method can generate a more accurate estimation model of respiratory movement and ensure safer operation.

Bailiang Chen et al. [80] designed a digital motion sensor compatible with MR, with the accelerometer as the main component. The sensor can model and predict breathing movements to implement free-breathing MR imaging strategies. In motion modeling and prediction, a linear regression method is used to extract the motion model applied to predict the displacement field calculated from the new physiological data obtained during the scan. The verification step is completed by calculating the predicted movementfield by comparing the sports field with the scanned images. The sensor for the breathing movement during MR imaging problems provides alternative sensor solutions, and patients may improve the convenience of installation.

In 2016, based on multi-scale Monte Carlo simulations, Y. Zhang et al. [81] developed an organ-to-cell level method for estimating the radiobiological effects of clinical radiotherapy. At the cellular level, cumulative damage is calculated using a spectrum-based accumulation algorithm and a predefined cell damage database, which can be used to evaluate individualized radiobiological effects in radiation therapy.

In 2017, P. F. et al. [82] proposed a system composed of three-dimensional motion models created by real-time magnetic resonance imaging for cardiac and respiratory motion compensation. The cardiac information derived from the ECG and the respiratory information extracted from the image are taken into account, multiple sagittal slices are stacked into a consistent three-dimensional volume, and the temporal smoothness of the stack is enhanced through the energy minimization formula. In addition, deformable 3D/3D registration is used to estimate motion from the magnetic resonance volume. The motion model itself is a linear direct correspondence model that uses the same alternative signal as the slice stack. Regarding the X-ray perspective, only the alternative signals need to be extracted to apply the motion model and display the overlay layer in real time.

In 2019, using robust principal component analysis (RPCA) and non-rigid image registration, C. M. Scannell et al. [83] proposed a fully automatic imaging method to achieve motion compensation of free-breathing perfusion MRI image sequences. RPCA allows for the dynamic contrast enhancement in myocardial perfusion CMR image sequences to be separated from baseline signals, and the deformation field used to eliminate respiratory motion can also be calculated in the absence of local contrast enhancement. In addition, the group registration method eliminates the difficulty of selecting reference frames.

In 2020, the combination of MRI scanners and linear accelerators enabled radiation therapy planning of internal organ movements estimated from MRI data. During radiation therapy, optimal MR guidance requires a delay of 200 to 500 milliseconds in MR-based 3D motion estimation. To address the problem of estimating organs in real time from MRI data, Niek R. F. et al. [84] proposed MR-MOTUS, which is a framework for estimating non-rigid three-dimensional motion from minimum K-space data. The framework consists of two main components: (i) a signal model that explicitly relates the K-space signal of a deformed object to a non-rigid sports field and a reference image, and (ii) model-based direct reconstruction of non-rigid sports fields from K-space data. By referring to the high spatial correlation between the image and the motion of the internal volume, the sports field is represented as a low-dimensional space, which can be reconstructed from the minimum K-space data. MR-MOTUS can reconstruct non-rigid three-dimensional respiratory movements in vivo from 63-fold retrospective undersampled K-space data. A real-time low-rank MR-MOTUS method can perform non-rigid 3D breathing motion estimation within a 170-millisecond delay, including acquisition and reconstruction.

With the demand for an ultra-high magnetic field (UHF), S. Dietrich et al. [85] from the Physikalisch-Technische Bundesanstalt (PTB) studied a 3D relative B_1_^+^ mapping sequence based on radial phase coded (RPE) K-space trajectories at 7 T. The method is based on a fast, low-power relative B_1_^+^ mapping and radial phase coding (RPE) acquisition method that allows for the retrospective grouping of respiratory motion states. The results of B_1_^+^ mapping between dynamic measurement and static reference acquisition were in good agreement.

#### 3.2.3. Ultrasound Imaging

**Figure 4:**
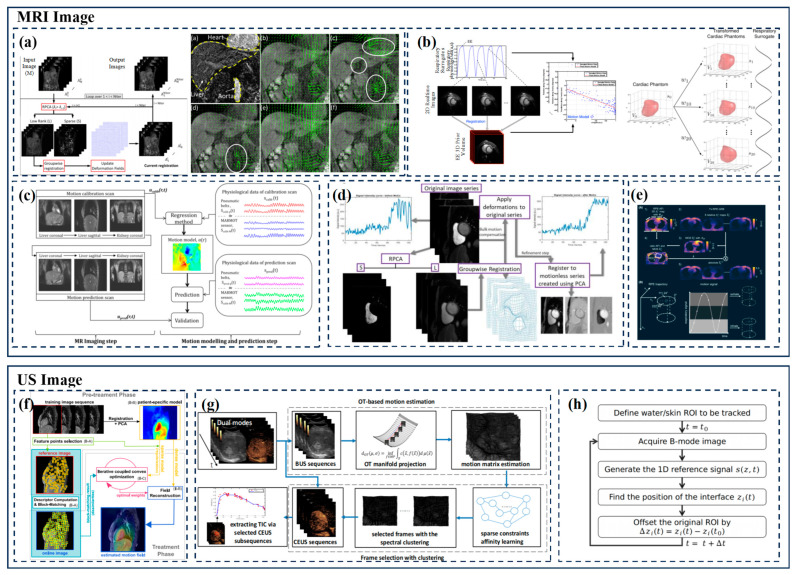
Overview of indirect model respiration prediction based on MRI and ultrasound images: (**a**) dynamic MRI respiratory motion correction based on robust data decomposition registration; (**b**) MRI-guided correction of intracardiac interventions based on respiratory motion model; (**c**) novel MR-compatible sensor for respiratory motion modeling and correction; (**d**) free-breathing myocardial perfusion MRI data of non-rigid motion compensation; (**e**) human 3D free-breathing multi-channel absolute B1+ mapping at 7 T; (**f**) based on the model of sparse to dense image registration in the MRI images to guide intervention respiratory motion estimation in real time; (**g**) transmission-based liver ultrasound compensation for irregular respiratory motion; (**h**) respiratory motion compensation of a robotic arm based on ultrasound images during extracorporeal boiling section of abdominal tissue.

The overview of indirect model respiration prediction based on MRI and ultrasound images is shown in Figure 4.

Ultrasound is an inexpensive, flexible, real-time imaging method with high temporal and spatial resolution (sub-millimeter spatial resolution in the plane along the beam direction). The high penetration of ultrasound in soft tissue during treatment can replace motion tracking by sensors. The first attempt at ultrasound-based motion tracking was to detect periodic and small-amplitude rigid motion using continuous one-dimensional ultrasonic echoes parallel to the direction of the main axis of motion. However, the local motion of the organs in the chest and abdomen is three-dimensional, and one-dimensional projection alone is not enough. Therefore, some studies looked at using both two-dimensional ultrasound and magnetic resonance imaging. Tretbar et al. [86] used a biplanar ultrasonic imaging transducer to broaden the field of view by allowing for a large angle of light beam steering. In recent years, there has been increasing interest in motion-tracking dynamic imaging due to its ability to capture the 3D deformation of the target. A 4D ultrasound can also be used to generate patient-specific models as preparation for intervention [87,88]. Unlike MR and CT, 4D ultrasound can be used for real-time motion tracking for local ablation therapy [33].

Ultrasound imaging, as a safe and radiation-free navigation protocol, is increasingly used in various surgeries. J. Zhang et al. [89] proposed an adaptive ultrasound scanning system (SAUSS) to image the human spine, automatically scanning the human back and displaying the spine structure in real time. Robotic 4D ultrasound (US) imaging systems capable of simultaneous radiotherapy beam delivery were described [35].

Using contrast invariant feature descriptors, I. Y. Ha et al. [90] proposed a method suitable for imaging modes with real-time capabilities (such as MR-Linac scanners and 3D-US). The method combines GPU-accelerated image-based real-time tracking of sparse distributed feature points with a dense patient-specific motion model, as well as sparse-to-dense interpolation and regularization in a unified optimization framework. The results show that the method can more realistically simulate physiological breathing movements, achieving highly accurate movement prediction using MRI (about 1 mm) and US (about 2 mm).

In 2020, in order to solve the problem of liver movement irregularity, P. Wan et al. [18] proposed a method for liver CEUS analysis of respiratory compensation based on movement estimation (RCME). This method uses the framework of optimal transport (OT) to clearly model the continuous change in the spatial distribution of tissue in the B-mode sequence, captures the expansion/contraction of local tissue by mapping, and realizes tissue matching and displacement estimation. Then, using the multi-subspace structure of the sequential motion matrix, the sparse subspace clustering (SSC) is used to identify CEUS subsequences corresponding to reference points to recover TIC.

In boiling histotripsy treatments, Gilles P. L. et al. [36] used a robotic arm to compensate for the active one-way breathing motion and an inline ultrasonic imaging probe to follow the movement of the skin to achieve an autoregressive motion prediction model in 2021.

#### 3.2.4. Others

In addition to traditional medical image data, in order to better study the mapping relationship between body surface respiratory movement and the target region in vivo, many research teams have carried out research on movement models based on structured light or infrared images.

In 2015, Z. Liang et al. [91] from Rensselaer Polytechnic Institute proposed a preoperative 4D shape derived from patient-specific breathing patterns to drive intraoperative range imaging (RI)-based real-time respiratory movement analysis. The information is encoded in a surface motion model that obtains 3D body surface data at different breathing states through non-rigid registration, and the patient’s current body surface obtained through multi-view RI registration. The information is encoded in a surface motion model that obtains 3D body surface data at different breathing states through non-rigid registration, and the patient’s current body surface is obtained through multi-view RI registration. During surgery, the motion model is registered to the patient via multi-view magnetic resonance imaging. Then, control is registered on the body surface. The framework supports the reconstruction of the dense body surface displacement field caused by respiration to generate custom respiratory substitutes.

In 2020, based on the work of chest and abdomen surface dynamic voxel modeling, S. Yu et al. [92] from Soochow University proposed a respiration movement characterization method based on chest and abdomen voxel modeling. The 3D modeling of the surface during chest and abdominal breathing was achieved using point cloud data from the depth camera. A dimensionality reduction algorithm was used to extract respiratory features from the voxel model, and a correlation model of tumor movement was established.

In 2021, to ensure that the correlation between external features extracted by skin surface movement and tumor movement varies in different regions, J. Wang et al. [93] proposed a method based on selecting moving surface areas with a high Pearson correlation coefficient with tumor movement. The surface region was divided into several regions, and an improved correlation model based on minimum cost function was proposed, taking into account the multi-dimensional structure of basic motion features. The experimental results show that the average and absolute errors of this model were smaller than those of the model using surface mark motion.

In 2022, to solve the dilemma between real-time and accurate estimation, Y. Shi et al. [94] combined holographic AR with digital twin technology to track dynamic surgical scenes through internal motion prediction and provide three-dimensional navigation of heterogeneous target areas, compensating for the time costs caused by external/internal correlation models and data transmission.

### 3.3. Respiratory Prediction Method Based on Indirect Learning

**Figure 5:**
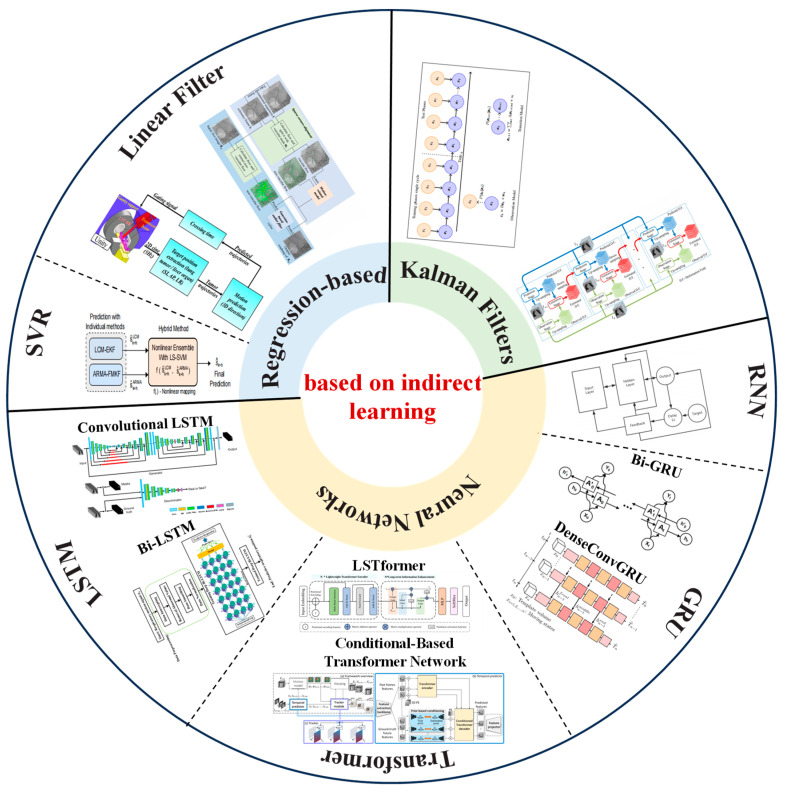
Overview of learning-based respiratory prediction methods.

Using respiratory motion prediction to overcome system delay can improve accuracy, and thus, many studies proposed learning-based respiratory motion prediction methods to improve the prediction accuracy of respiratory motion. Seregni et al. [95] proposed a neural network model for phantom testing to demonstrate the feasibility of real-time tumor tracking based on external respiratory signals. With the continuous development of deep learning, learning-based respiratory motion can be predicted by filters (such as linear filters and Kalman filters) and neural networks. This section is mainly divided into regression-based methods, Kalman filters, and neural networks [96]. The overview of learning-based respiratory prediction methods is shown in Figure 5.

#### 3.3.1. Regression-Based Methods

Regression-based methods mainly include linear regression models, multi-scale wavelet autoregression, an autoregressive integrated moving average (ARIMA) model, support vector regression (SVR), and the least squares method [97].

In recent years, several techniques were proposed to predict respiratory movement. This includes local circular motion (LCM), kernel density estimation (KDE), and support vector regression (SVRpred) [98]. Most of the prediction techniques were compared and analyzed by relevant studies. Among them, LCM-EKF shows better estimation ability and lower computational complexity, while the performance of LCM-EKF tends to have larger prediction lengths (e.g.; 400 ms) and irregularities in the subjects’ breathing patterns [99]. To overcome the delay of radiation machines in treating lung tumors, S. Tatinati et al. [99] introduced a hybrid method based on a least squares support vector machine (LSSVM) for predicting respiratory movement. The method utilizes the comparative advantages of various methods, namely, local circular motion (LCM), extended Kalman filter (EKF), autoregressive moving average (ARMA) model, and attenuation memory Kalman filter (FMKF). The results show that the method improved the prediction accuracy by about 10% in a prediction time of 460 milliseconds compared with existing methods in 2014.

In 2018, N. K. Y. et al. [100] developed a stage oscillator model that predicts the location of lung tumors based on previous data of lung tumors, current breathing waveforms, and heartbeat measurements. The results show that the RMS error of the estimated tumor location provided by the model was 1.5 mm when simulated offline.

To verify the effectiveness of linear regression in predicting internal organ deformation or tumor movement in 2D dynamic magnetic resonance imaging (cine-MR), Y. Li et al. [101] proposed an online gated signal prediction scheme that can improve the accuracy of MRI-guided radiotherapy for liver and lung cancer in 2023. This method uses a binary gated signal prediction algorithm to predict the cross time of the tumor trajectory relative to the target threshold. The results show that the amplitude errors of this method were significantly reduced compared with those of an RNN in the cases of 0.6 s and 0.4 s predictions.

Blood vessels constantly move/deform due to respiratory movements and are not visible in X-ray images unless injected with contrast agents. K. Yang et al. [102] proposed a vascular respiratory motion compensation algorithm (MRC) based on optical flow, which compensates for vascular respiratory motion by inferring the correlation between invisible blood vessels and visible non-blood vessels. In robot-assisted image-guided intervention, the method can predict 2D vascular road maps in real-time X-ray images. After the injection of a contrast agent, the vascular respiration motion compensation was performed based on a sparse Lucas–Kanade feature tracker. The MRC model is trained to learn correlations between vascular and non-vascular movements, predicts invisible blood vessels using the visible tissue and MRC model, and is refined with a Gauss-based outlier filter. The method could achieve vascular respiration motion compensation in 0.032 s with an average error of 1.086 mm.

#### 3.3.2. Kalman Filters

The prediction of respiratory motion based on a Kalman filter is the optimal estimation of system state based on observation data. Previous studies constructed filters, such as a Kalman filter or extended Kalman filter [97].

In 2018, R. L. Smith et al. [103] formulated the estimation of respiratory motion under the hidden Markov model (HMM), constructing a Kalman filter using the motions extracted from dynamic images of a single respiratory cycle and their associated observed signals. The EM algorithm is combined to find maximum likelihood estimates of HMM parameters on a per cycle basis given a set of observations or external alternative signals. The method also uses PCA on external alternative signals and parametric internal motions, respectively, thus providing a basis for projecting them onto two different low-dimensional manifolds. The expectation maximization Kalman filter performs parameter estimation and adaptively adjusts the respiration estimation of irregular motion. This method has three advantages: (1) motion estimation can be performed even when the patient’s breathing pattern is different from that observed during training; (2) changes in model noise and observed or external alternative signal noise are taken into account; and (3) the degree of fit of the underlying model can be parameterized to determine the confidence of the accuracy of the correction method.

For dynamic motion estimation of continuous phases, in 2020, P. Xue et al. [104] proposed a lung respiratory motion estimation method (LRME-4DCT) based on fast Kalman filtering and 4DCT image registration. Each phase was registered using isoPTV and HOMRF registration methods, and the registration results were used as the observation and prediction vectors of the constructed motion estimation model, respectively. In order to solve the high computational requirements of 4DCT image sequences, LRME-4DCT adopts a multi-level processing strategy to predict the breathing motion state from three directions. Compared with traditional estimation methods based on paired image registration, the LRME-4DCT method can estimate physiological respiratory movement more accurately and quicker.

M. Frueh et al. [105] introduced a framework for landmark detection and tracking and proposed an adaptive patient-specific respiratory movement model for parameter estimation by an expectation-maximization Kalman filter. The method introduces a generalization capability that allows for adaptive adjustments in the presence of irregular motion, enabling the model to consider a wider range of motion changes. This method is based on the least labeled data for real-time landmark detection with self-supervised training. Unlike the self-supervised method that relies on the similarity measure of image blocks, this method is embedded from the local to global position, which enables computationally efficient prediction. The usefulness of the method was demonstrated by using it for automatic real-time liver lesion tracking in time-resolved abdominal magnetic resonance imaging (MRI) and for real-time automated liver tracking on magnetic resonance linear accelerator (MR-LINAC) data and routine chest X-rays.

**Neural Networks**

The latest progress in machine learning technology has improved the quality of medical images and promoted the in-depth research of artificial intelligence (AI) in the field of medical image analysis. Based on a branch of artificial intelligence, deep learning has been successfully applied to problems such as image classification or segmentation, including tumor detection and segmentation on medical images. In respiration prediction, ANN, RNN, LSTM, and other network architectures are also used to predict the motion trajectory.

In 2015, I. Bukovsky et al. [70] used a classical linear model, a perceptron model, and a class of higher-order neural network models to investigate the real-time prediction of a 3D time series of lung tumor movements. The results show that the prediction accuracy of 1 mm 3D MAE in the prediction range of 1 s is much shorter than the actual processing time.

In 2017, W. Sun et al. [106] used adaptive augmentation and multi-layer perceptron neural networks (ADMLP-NNs) to predict respiratory signals. An ADMLP-NN consists of multiple artificial neural networks (ANNs) that are used as weaker predictors to combine into a stronger predictor. The breathing signal is first smoothed using the Savitzky–Golay finite impulse response smoothing filter (S-G filter). For 500 milliseconds of prediction, the average correlation coefficient was improved from 0.83 (MLP-NN method) to 0.89 (ADMLP-NN method). Compared with the case with the MLP-NN, the RMS error (in relative units) of the 500 ms prediction was reduced by an average of 27.9% using the ADMLP-NN.

In 2018, J. Kai and F. Fujii et al. [107] from Yamaguchi University proposed a model that uses recurrent neural networks (RNNs) to predict three-dimensional tumor movement. Compared with traditional neural networks, an RNN has the abilities of persistence and memory, which are suitable for modeling discrete-time dynamic systems. Because the motion is three-dimensional, three recurrent neural networks are used to predict the motion trajectories of x-, y-, and z-axes. The root-mean-square error (RMSE) of the predicted trajectory was less than 1 mm within 1 s of the prediction time.

Regarding rotational angiography, M. Unberath et al. [7] investigated two a priori respiratory motion estimation methods based on polar-line consistency condition (ECC) optimization and task-based autofocus metric (AFM) for the estimation of coronary artery respiratory motion in rotating coronary angiography. When ECC- and AFM-based compensation were applied, the vascular clarity was improved by 6.08 ± 4.46% and 14.70 ± 8.80%, respectively. Using CAVAREV data, the ECC and AFM methods improved 27.6 ± 7.5% and 97.0 ± 17.7%, respectively. Both of these motion estimation strategies are purely image based and are able to accurately estimate the displacement of the coronary arteries due to breathing.

One-way LSTM preserves only past information, while bidirectional LSTM preserves past cell passes and state information from the future, and performs better for sequential problems [108]. The research team at the Shenzhen Institutes of Advanced Technology proposed a seven-layer bidirectional short-duration memory network (Deep Bi-LSTM) and a deep neural network with an output layer for predicting breathing movements in about 400 milliseconds. The results show that the prediction accuracy of Deep Bi-LSTM was about five times better than that of the traditional autoregressive integral moving average (ARIMA) model, and about three times better than the adaptive lift and multi-layer perceptron neural network (ADMPL-NN) with a delay of 400 ms [109].

In addition, in order to solve the problem where LSTM networks take a long time to update and may not be able to update the prediction model within a single X-ray acquisition cycle, S. Yu et al. [97] proposed a fast prediction model based on a Bi-gated cycle unit (Bi-GRU). A GRU is a simplified version of an LSTM with simple structure and no loss of basic feature information of data series in the prediction process. This method can reduce the average update time of the network model by 30%.

In 2021, L. V. R. et al. [110] proposed a population-based generation network to solve the problem of predicting the position of a three-dimensional target from a two-dimensional image-based alternative during radiation therapy, enabling treatment target tracking using images acquired in real time. Due to the powerful generalization ability of neural networks, the model does not need to establish correspondence between subjects and can be quickly deployed in just 8 milliseconds of inference time. The training model is represented by a three-dimensional low dimensional manifold with non-rigid deformation. The predictive power of the model can correct errors in target placement that may occur due to system delays when using only the baseline volume of the patient’s anatomy. In addition, the method does not require supervisory information, such as ground-truth registration sites, organ segmentation, or anatomical markers.

In 2022, M. Tan et al. [111] proposed a long- and short-term transformer (LSTformer) for accurate prediction of real-time breathing under long windows. The method uses lightweight transformer encoder (LTE) to simulate the semi-periodic time dependence of breathing signals to meet real-time requirements. In addition, the long-term information enhancement (LIE) module in the method can enhance the long-term memory of latent variables encoded by a lightweight transformer to solve the performance degradation problem in long window prediction. The method also proposes an application-oriented data enhancement strategy (AOA), which solves the problem where existing public data sets cannot fully cover practical application scenarios and demonstrates the diversity of public data sets collected by optical trackers. The depth camera collects new data on the simulator to train the model, thereby improving the generalization ability of the model.

T. Peng et al. [112] captured the consistent movement of tumors in fluoroscopic images through a neural-network-based model that is trained using generative adversarial methods. The network adopts coarse-to-fine architecture design and introduces a convolutional long short-term memory (LSTM) module to consider the temporal correlation between different frames of perspective images. The model was trained and tested using a digital X-CAT phantom. To fully evaluate the accuracy of the model, phantoms of different scales, tumor locations, sizes, and breathing amplitudes were generated. The results showed that the mean IOU and Dice coefficients were 0.93 ± 0.04 and 0.96 ± 0.02, respectively; the mean tumor AD was 4.34% ± 4.04%; and the mean COMD in the upper and lower (SI) and left and right (LR) directions were 0.16 and 0.07 cm, respectively. To investigate the effect of motion amplitude on tracking accuracy, phantoms with fixed body and tumor sizes but different breathing amplitudes were generated, and the results showed that the mean IOU and Dice coefficients reached 0.98 and 0.99, respectively, with a mean tumor difference of 0.17%. In the SI and LR directions, the average COMDs were 0.03 and 0.01 cm, respectively.

In 2023, L. V. R. et al. [113] proposed an attention-based time prediction network that treats features extracted from input images as markers of prediction tasks. The network architecture consists of three modules: feature coding and decoding, conditional transformer network, and parallel prior-based latent modeling. This method can simultaneously learn to map the dense deformation between image pairs and extrapolate through time by using a set of learnable queries to predict potential representations of future deformations, conditioned on prior knowledge. Compared with the condition-based 4D transformer motion model, the error of this model was reduced by 63%, with an average error of 1.5 ± 1.1 mm.

Based on the idea of dense connected convolutional networks (DenseNet), M. Bengs et al. [91] proposed an efficient 4D architecture that can process long-term 4D ultrasound sequences in real time. According to the parameter efficiency and feature propagation intensity of DenseNet, 3D mode is used for the spatial processing of volume ultrasonic data. The method uses ConvGRUs recurrent neural network processing time for 2D image sequences. Considering the different scales and shapes of the target sites following respiratory movement during radiotherapy, this method analyzes the spatio-temporal characteristics of different scales for the movement during radiotherapy and proposes the spatio-temporal circulation at different feature levels.

## 4. Discussion

In the face of the increasing number of tumors and the high mortality rate of cancer around the world, the study of intraoperative respiratory movement monitoring and prediction in the chest and abdomen will become a relatively advanced and hot field for further exploration in the next few decades. Currently commercially available respiratory movement compensation methods for therapeutic target areas remain a key and difficult issue in this field. According to the summary of this paper, respiratory monitoring and compensation methods still have a lot of room for improvement and improvement in future research:

**(1).**  **Respiratory movement tracking without markers:**

By means of optical and radar monitoring combined with Linchuan medical theory, a non-invasive and non-contact respiratory movement tracking system for the chest and abdomen was realized. The redundant radiation dose was reduced when guided by CT images during the tracking process, and the probability of radiation causing damage or lesions to normal tissues or organs was also reduced.

**(2).**  **Guidance technology combined with ultrasonic imaging:**

Ultrasound is an inexpensive, flexible, real-time, and radiation-free imaging method with high temporal and spatial resolution, i.e.; submillimeter spatial resolution in the plane along the direction of the beam. Using the high penetration of ultrasound in soft tissue, movement can be tracked without the use of sensors during treatment. However, since the external ultrasonic imaging probe cannot send a beam parallel to the axis of respiratory motion, the local organ motion is space-dependent, and using only one-dimensional projection is not enough. Digital phantoms can also be constructed with 4D ultrasound using a biplanar ultrasonic imaging transducer that allows for the beam to be turned at a large angle, giving the organ a wider field of view at depth. Unlike MR and CT, 4D ultrasound can be used not only for real-time motion tracking but also for local ablation therapy. Future studies should explore the applicability of ultrasound imaging for breathing tracking.

**(3).**  **Combined deep learning and respiration prediction model construction:**

The respiration prediction based on the prior collection of sequential images cannot accurately predict the respiration movement that is not included in the existing sequential images. Through deep learning technology, the steps related to image registration and feature point detection can be reduced, and breathing tracking can be fully automated, reducing the workload of researchers. However, for image-based neural network prediction models, the computer configuration requirements are high, and having a large number of models also means a long delay. Direct input of the collected raw data may cause data redundancy and increase the burden of the model. The rapid and effective prediction of deep learning models can be achieved through dimensionality reduction, principal component analysis, and other technologies.

To address the difficult tradeoffs between safety, real time, and accuracy in clinical applications, we identified three future directions. These directions are of great significance for improving respiratory motion compensation in existing chest and abdominal treatments.

## 5. Conclusions

The purpose of this review was to systematically review the application of respiratory movement monitoring and prediction in the diagnosis and treatment of the chest and abdomen. According to the different compensation methods, the recent development status of medical imaging combined with sensors (such as optical and acoustic sensors) or deep neural network in respiratory movement tracking is briefly summarized through three aspects. It is hoped that this will be helpful to researchers in this field. This review summarizes the techniques in this paper in a systematic manner. Specifically, we provide an overview of chest and abdominal respiratory movements and compensation in recent years of treatment in the reviewed literature. Finally, we discuss the future development of monitoring and prediction methods for chest and abdominal respiration. The respiratory monitoring and compensation methods need to be further improved in future studies. First of all, the non-invasive and non-contact tracking of the chest and abdomen was realized through the unmarked respiratory movement tracking system, combined with optical and radar monitoring and clinical medical theory, which reduces the radiation dose guided by CT images and reduces the risk of radiation damage. Second, combined with the guidance technology of ultrasound image, the tracking of soft tissue movement was realized by using the high penetration of ultrasound. In particular, the field of vision in depth is expanded by constructing a four-dimensional ultrasonic digital model, which provides the possibility for real-time motion tracking and local treatment. Finally, combined with deep learning technology and the construction of a respiratory prediction model, breathing tracking can be automated, reducing the work burden of researchers. However, it should be noted that the image-based neural network prediction model may have problems, such as high computer configuration requirements and a long delay. The prediction efficiency of the model can be optimized by a dimensionality reduction, principal component analysis, and other techniques. Although research articles mentioned a variety of methods, some articles explain the comprehensiveness and feasibility of these methods and room for improvement. To be specific, most of the studies related to unmarked respiration tracking method were only in the experimental stage, lacking details of application in actual clinical environment. In the future, this method can be verified in clinical practice to carry out specific studies. Future studies should explore further applications and refinements of these methods to improve the efficacy and safety of respiratory motion compensation in therapeutic target areas. This article will help researchers to understand the recent progress of respiratory monitoring and prediction in the chest and abdomen.

## Figures and Tables

**Table 1:** Overview of tracking strategies for respiratory motion.

Group	Tracking Strategy	Representative Works	Characteristics
Contact	Noise sensor: noise variance based on RF coil	A. Andreychenko et al. [20], J. M. Navest et al. [21]	(1) No need for careful positioning or any additional hardware; (2) Combined with Kalman filtering, respiratory signals can be extracted and predicted without delay; (3) Can measure breathing passively, independent of MR signal; (4) Some limitations in temporal resolution and spatial resolution.
	RGB-D camera with markers	U. W and S. P. et al. [22], Y. Yu et al. [8], M. Musa et al. [23]	(1) The system setup is very simple, very flexible, and portable; (2) Will not interfere with the patient‘s breathing, non-invasive benchmark marking, shortens the treatment time, and high safety; (3) The surface positioning accuracy is high, which can reach the millimeter level; (4) The performance is easily disturbed by factors such as light, background, and occlusion; (5) The camera needs the right position and angle.
	Electromagnetic sensor	Esther N. D. Kok et al. [24]	(1) Real-time and accurate tumor location information and key anatomic information can be obtained, which may reduce the occurrence of positive resection margins and improve the patient prognosis; (2) High tracking accuracy for targets in vivo; (3) Susceptible to electromagnetic interference, not suitable for MR.
	Pressure sensor	T. Addabbo et al. [25], H. L. et al. [26], Anthony L. et al. [5]	(1) Other invasive devices can be avoided; (2) It has the potential to be applied in 4D dose calculation to remove respiratory motion artifacts in positron emission tomography (PET) or γ scintillation image reconstruction; (3) The measurement accuracy is relatively high; (4) Accuracy is affected by its installation location; (5) Some patients may not be able to adapt to the pressure of the sensor; (6) Prolonged use may cause performance degradation or damage.
	Fiber Bragg grating sensors	C. M. et al. [27], C. Shi et al. [28]	(1) Comfortable and easy to wear, will not cause discomfort to the wearer; (2) Can be used in an MR environment; (3) No image artifacts are generated; (4) It has high sensitivity and enables simultaneous and accurate measurement of respiratory and cardiac activity; (5) Installation and maintenance are complicated; (6) High cost compared with some other sensors; (7) Sensitive to environmental conditions.
Non-contact	DC coupled CW radar sensor	C. Gu et al. [29]	(1) Non-contact and non-invasive; (2) Can accurately measure the movement, where the measurement accuracy can reach sub-millimeter level; (3) It has great potential in adaptive radiotherapy; (4) Relatively complex system; (5) High cost compared with some other sensors.
	RGB-D camera without markers	Shi H. Lim, P. Hou et al. [30], Andrew L. Fielding et al. [31], L. Zheng et al. [32]	(1) The system setting is very simple, very flexible, and portable; (2) Will not interfere with the patient’s breathing, non-contact and non-invasive, shortens the treatment time, and high safety factor; (3) The accuracy of surface positioning is higher, but may be lower than that of a system with markers.
	Directly image-guided	S. Vijayan et al. [33], L. R. et al. [34], J. S. et al. [35], Gilles P.L. et al. [36]	(1) The system has high detection accuracy and good applicability and can track the internal target movement in real time; (2) May cause unnecessary radiation to patients.

## Data Availability

The data used to support the findings of this study are available from the corresponding author upon request.
